# Bloqueio de Ramo Alternante a Cada Batimento

**DOI:** 10.36660/abc.20230162

**Published:** 2023-12-19

**Authors:** Margarida Temtem, Joel Ponte Monteiro, Marco Gomes Serrão, Drumond Freitas

**Affiliations:** 1 Hospital Dr. Nélio Mendonça Funchal Portugal Hospital Dr. Nélio Mendonça, Funchal – Portugal

**Keywords:** Arritmias Cardíacas, Eletrocardiografia/métodos, Bloqueio de Ramo, Bloqueio Atrioventricular, Hospitalização, Marca-passo Artificial

**Figura 1:**
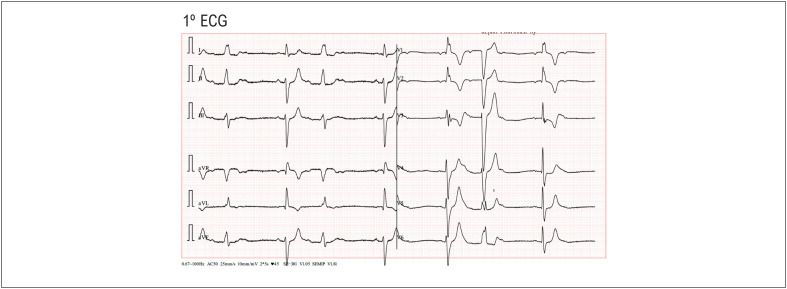
Bloqueio de Ramo Alternante (alternância a cada batimento de BRD e BRE), com BAV de 2º grau tipo Mobitz I.

Homem de 67 anos admitido no serviço de urgência dos cuidados de saúde primários por tonturas. Ao exame físico foi detectado um ritmo cardíaco irregular e pedido um eletrocardiograma (ECG) de 12 derivações. O ECG (Figura 1) mostrou um padrão de bloqueio de ramo alternante, com alternância a cada batimento de bloqueio de ramo direito e esquerdo (BRD e BRE) e com bloqueio atrioventricular (BAV) de 2º grau tipo Mobitz I.

**Figura 2:**
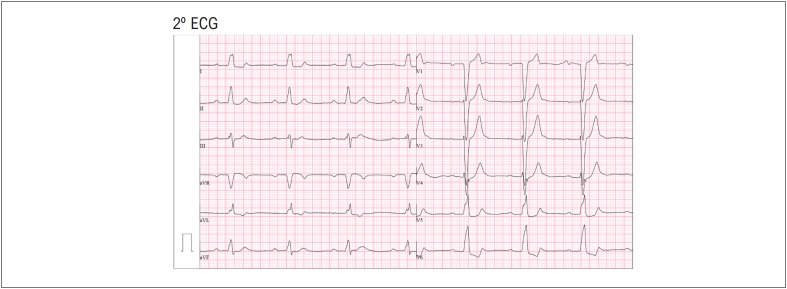
BRE persistente com BAV de 1º grau (intervalo PR de 320ms).

**Figura 3:**
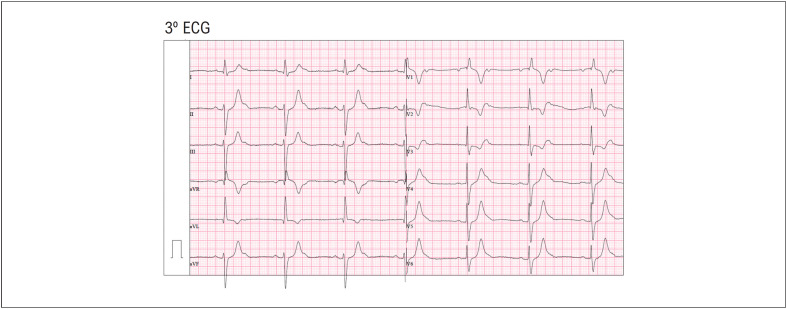
BRD persistente com bloqueio fascicular anterior esquerdo e BAV de 2º grau tipo

O paciente foi transferido para o serviço de urgência do hospital onde repetiu dois ECG sequenciais. Um ECG (Figura 2) com BRE persistente com BAV de 1º grau (intervalo PR de 320ms) e outro ECG seguinte (Figura 3) que mostrou um BRD persistente com bloqueio fascicular anterior esquerdo e BAV de 2º grau tipo 2:1 fixo.

Os dois ECG sequenciais, realizados poucos minutos depois, complementaram o diagnóstico do 1º ECG (Figura 1) que mostrava alternância a cada batimento entre BRD e BRE com BAV 2º grau tipo Mobitz I. Como demonstrado neste caso clínico, este bloqueio da condução intraventricular de modo alternante pode ocorrer de forma intermitente ou persistente. O mecanismo exato por detrás de um bloqueio alternante nem sempre é totalmente compreendido, podendo estar relacionado com anormalidades do Sistema His-Purkinje; associado a doença cardíaca estrutural, como fibrose ou cicatriz no sistema de condução; como também a outros mecanismos que podem ser mais ou menos comuns.^1^

Conforme observado, existe um atraso de condução variável em ambos os ramos ventriculares, que podem ser explicados pelos diferentes períodos refratários.^2^ Neste caso, quando o PR é mais curto, o estímulo elétrico desce pelo ramo esquerdo (padrão BRD) e, quando o PR é mais longo, o estímulo desce pelo ramo direito (padrão BRE). Adicionalmente, o atraso ou bloqueio do nódulo AV também varia, particularmente com a variabilidade da frequência auricular subjacente, sendo o grau de BAV mais avançado em frequências auriculares mais elevadas. Assim, nestes três eletrocardiogramas são visíveis BAV de diferentes graus: de 2º grau tipo Mobitz I no 1º ECG (Figura 1); BAV 1º grau no 2º ECG (Figura 2) e BAV de 2º grau tipo 2:1 fixo no 3º ECG (Figura 3).

O registo eletrocardiográfico de bloqueio de ramo alternante é raro na prática clínica,3 sendo ainda mais invulgar encontrar este padrão no mesmo ECG, com alternância de condução a cada batimento ventricular e, adicionalmente, com BAV. Alguns autores afirmam que o bloqueio de ramo alternante constitui cerca de 6% de todos os bloqueios de ramo e o maior receio deste distúrbio do ritmo é o risco potencial de BAV completo, sendo uma indicação classe I para implantação de marca-passo definitivo.^3,4^

Neste sentido, o paciente foi internado no serviço de Cardiologia tendo sido implantado um marca-passo definitivo de dupla câmara.
