# Supplementary material for: Bloqueio de Ramo Alternante a Cada Batimento
Source: Arq Bras Cardiol. 2023 Dec 19;120(12):e20230162. [Article in Portuguese] doi: 10.36660/abc.20230162 (PMC10735210; doi:10.36660/abc.20230162)
Supplement: Translation

# Translation: Beat-to-Beat Alternating Bundle-Branch Block

**Authors:** Margarida Temtem, Joel Ponte Monteiro, Marco Gomes Serrão, Drumond Freitas

**Affiliations:** 1 Funchal Portugal Hospital Dr. Nélio Mendonça, Funchal – Portugal

**Keywords:** Arrhythmias, Cardiac, Electrocardiography/methods, Atrioventricular Block, Hospitalization, Permanent Pacemaker

A 67-year-old man was admitted to the primary healthcare emergency department due to dizziness. During the physical examination, an irregular heart rhythm was detected, prompting a request for a 12-lead electrocardiogram (ECG). The ECG (Figure 1) revealed a beat-to-beat alternating pattern of right and left bundle branch blocks (RBBB and LBBB) with Mobitz I second-degree atrioventricular (AV) block.

**Figure 1:** Alternating Bundle Branch Block (beat-to-beat alternation between RBBB and LBBB), with Mobitz I second-degree AV block.

The patient was transferred to the hospital emergency department, where two consecutive ECGs were performed. One ECG (Figure 2) showed persistent LBBB with first-degree AV block (PR interval of 320ms), and the subsequent ECG (Figure 3) indicated persistent RBBB with left anterior fascicular block and fixed 2:1 second-degree AV block.

**Figure 2:** Persistent LBBB with first-degree AV block (PR interval of 320ms).

**Figure 3:** Persistent RBBB with left anterior fascicular block and fixed 2:1 second-degree AV block.

The two sequential ECGs, performed at a few minute intervals, complemented the diagnosis of the first ECG (Figure 1), which showed a beat-to-beat alternation between RBBB and LBBB with Mobitz I second-degree AV block. As demonstrated in this clinical case, this alternating intraventricular conduction block can occur intermittently or persistently. The exact mechanism behind alternating block is not always fully understood; it might be related to abnormalities in the His-Purkinje system, associated with structural heart disease, such as fibrosis or scarring in the conduction system, as well as other mechanisms that may be more or less common.^1^

As depicted in this case, there is a variable conduction delay in both ventricular branches, which can be explained by different refractory periods.^2^ In this case, when the PR interval is shorter, the electrical stimulus descends through the left branch (RBBB pattern), and when the PR interval is longer, the stimulus descends through the right branch (LBBB pattern). Additionally, the delay or blockage of the AV node also varies, particularly with underlying atrial frequency variability, with more advanced AV block at higher atrial frequencies. Hence, these three electrocardiograms display varying degrees of AV block: Mobitz I second-degree AV block in the first ECG (Figure 1), first-degree AV block in the second ECG (Figure 2), and fixed 2:1 second-degree AV block in the third ECG (Figure 3).

The electrocardiographic recording of alternating bundle branch block is rare in clinical practice,3 and it is even more unusual to find this pattern in the same ECG, with beat-to-beat alternating conduction and, additionally, with AV block. Some authors state that alternating bundle branch block constitutes approximately 6% of all bundle branch blocks, and the primary concern with this rhythm disturbance is the potential risk of complete AV block, which is a Class I indication for permanent pacemaker implantation.^3,4^

Therefore, the patient was admitted to the Cardiology department and underwent the implantation of a dual-chamber permanent pacemaker.
